# Li-Fraumeni Syndrome: Current Perspectives on Molecular Pathogenesis, Surveillance Strategies, Clinical Management and Pediatric Considerations

**DOI:** 10.7759/cureus.114575

**Published:** 2026-08-15

**Authors:** Gillian Graifman, Sophia Currie, Ranya Parekh, Rajesh Varma C P, Arun Nair

**Affiliations:** 1 Pediatrics, Cohen Children's Medical Center, New Hyde Park, USA; 2 Pediatrics, Donald & Barbara Zucker School of Medicine at Hofstra/Northwell, New Hyde Park, USA; 3 Medicine, Donald & Barbara Zucker School of Medicine at Hofstra/Northwell, Hempstead, USA; 4 Pediatrics, Saint Peter's University Hospital, New Brunswick, USA; 5 Pediatric Hematology Oncology, Cohen Children's Medical Center, New Hyde Park, USA

**Keywords:** cancer predisposition syndrome, " li-fraumeni syndrome", toronto protocol, tumor pathway, tumor suppressor protein p53

## Abstract

Li-Fraumeni Syndrome (LFS) is a rare autosomal dominant hereditary cancer predisposition syndrome caused by germline mutations in the TP53 tumor suppressor gene, conferring a markedly increased lifetime risk of multiple early-onset malignancies. Pediatric patients are particularly susceptible to developing soft tissue sarcomas, osteosarcomas, adrenocortical carcinomas, central nervous system tumors, and hematologic malignancies, necessitating early recognition and lifelong surveillance. Over the past decade, substantial advances in the understanding of TP53 biology have deepened insight into the molecular mechanisms underlying tumor development and informed the evolution of comprehensive surveillance strategies. This literature review summarizes the current understanding of the molecular pathogenesis of LFS, reviews the pediatric cancer spectrum, and compares contemporary surveillance recommendations, including the Toronto protocol, European Reference Network (ERN) GENTURIS guidelines, and recent American Association for Cancer Research (AACR) recommendations. We further discuss current clinical management, challenges associated with lifelong surveillance, and emerging therapeutic approaches, including mutant p53 reactivation, synthetic lethality strategies, and pharmacologic cancer prevention with agents such as metformin. Although no targeted therapy has been approved specifically for individuals with LFS, advances in precision medicine and ongoing clinical trials continue to expand the potential for preventive and therapeutic options. Continued international collaboration, prospective clinical research, and integration of molecular discoveries into clinical practice will be essential to optimize surveillance protocols, develop effective targeted interventions, and improve long-term outcomes for children and families affected by Li-Fraumeni Syndrome.

## Introduction and background

Li-Fraumeni syndrome (LFS) was first described in 1969 by Frederick P. Li and Joseph F. Fraumeni Jr., who identified familial clustering of childhood soft-tissue sarcomas, early-onset breast cancer, and other malignancies consistent with an autosomal dominant cancer-predisposition syndrome [1]. LFS is now understood to result primarily from germline pathogenic or likely pathogenic variants in the tumor protein p53 gene, TP53, which encodes the p53 tumor-suppressor protein [2]. Often referred to as the “guardian of the genome,” p53 functions as a stress-responsive nuclear transcription factor involved in cell-cycle arrest, DNA repair, senescence, and apoptosis [3]. Individuals with LFS are predisposed to a broad spectrum of malignancies, particularly soft-tissue and bone sarcomas, premenopausal breast cancer, central nervous system tumors, adrenocortical carcinoma, and hematologic malignancies [2].

Pathogenic germline variants in TP53, located on chromosome 17p13.1, represent the principal molecular basis of LFS [2]. These variants are constitutionally present in affected individuals and must be distinguished from somatic TP53 alterations, which are acquired within tumor cells and are not necessarily indicative of an inherited cancer-predisposition syndrome. Cancer risk in germline TP53 variant carriers is exceptionally high and begins in early childhood, although penetrance varies according to sex, variant type, family ascertainment, and other modifying factors [2,4]. In children, the characteristic tumor spectrum includes soft-tissue sarcomas, osteosarcoma, adrenocortical carcinoma, central nervous system tumors, and selected hematologic malignancies [2,4].

The distinction between germline and somatic TP53 alterations is particularly important in certain pediatric cancers. Biallelic TP53 alterations are present in most cases of low-hypodiploid acute lymphoblastic leukemia, and a substantial proportion of affected children have an underlying germline TP53 variant [5]. Pediatric adrenocortical carcinoma is also strongly associated with germline TP53 variants; however, reported prevalence varies markedly by population and is particularly high in regions of southern Brazil where the founder variant p.Arg337His is prevalent [6]. In a Children’s Oncology Group cohort, germline TP53 variants were identified in approximately half of tested children with adrenocortical carcinoma rather than in 80% of all pediatric cases [6].

Because malignancy risk begins in infancy and continues throughout life, structured surveillance is an essential component of LFS management. The European Reference Network for Genetic Tumour Risk Syndromes (ERN GENTURIS) recommends clinical examination every six months in children, abdominal ultrasonography every six months for adrenocortical carcinoma surveillance, annual brain magnetic resonance imaging, and annual whole-body magnetic resonance imaging [2]. The guideline also recommends minimizing exposure to ionizing radiation whenever clinically appropriate because of concern for treatment-related and radiation-associated subsequent malignancies [2].

The American Association for Cancer Research issued updated LFS surveillance recommendations in 2025. These recommendations retain intensive age-directed surveillance and include physical examination and abdominal and pelvic ultrasonography every three to four months during childhood, annual whole-body MRI, and annual brain MRI, together with additional organ-specific screening according to age and risk [7]. The AACR recommendations therefore should not be described as identical to the ERN GENTURIS guideline because several surveillance intervals differ.

Prospective surveillance studies led by Villani and colleagues provided important evidence supporting this approach. The initial prospective study was published in 2011, while the subsequent 11-year follow-up was published in 2016 [8,9]. In the extended follow-up, surveillance was associated with earlier tumor detection and improved five-year overall survival, estimated at 88.8% among individuals undergoing surveillance compared with 59.6% among those who did not undergo surveillance [9]. These findings established structured multimodal surveillance as a central component of contemporary LFS care (Table 1).

**Table 1:** Core pediatric surveillance recommendations for individuals with Li-Fraumeni syndrome Comparison of core pediatric surveillance recommendations derived from the Toronto surveillance studies, ERN GENTURIS guideline, and 2025 AACR consensus recommendations [2,7-9]. AACR: American Association for Cancer Research; ERN GENTURIS: European Reference Network for Genetic Tumour Risk Syndromes

Surveillance target	Suggested modality and interval
General assessment	Comprehensive physical examination every 3–4 months under the 2025 AACR recommendations; every 6 months under ERN GENTURIS
Adrenocortical carcinoma	Abdominal and pelvic ultrasonography every 3–4 months under AACR; abdominal ultrasonography every 6 months under ERN GENTURIS
Soft-tissue and bone sarcomas	Annual whole-body MRI
Central nervous system tumors	Annual brain MRI
Hematologic malignancies	Routine CBC-based screening is included in some Toronto-derived protocols but is not uniformly recommended across all contemporary guidelines
Skin cancer	Periodic dermatologic examination based on age and guideline framework
Breast cancer	Breast awareness beginning in adolescence and annual breast MRI in early adulthood; specific starting age depends on the guideline
Radiation exposure	Minimize ionizing-radiation-based screening and treatment when a clinically appropriate alternative exists

This review examines the molecular pathogenesis and pediatric cancer spectrum of LFS, compares contemporary surveillance recommendations, and evaluates current and emerging management strategies. Particular attention is given to the Toronto surveillance approach, ERN GENTURIS guidelines, and updated AACR recommendations, as well as to emerging molecularly directed and preventive therapeutic strategies.

## Review

Methods 

This study was conducted as a narrative literature review. The research questions were discussed and agreed upon by all members of this research team prior to initiation of the study, and article curation was completed using PubMed and Google Scholar. Inclusion criteria consisted of English-language articles, human studies, and studies relevant to Li-Fraumeni Syndrome, particularly those involving pediatric populations or findings applicable to pediatric clinical practice. Search terms included “Li-Fraumeni Syndrome”, “Pediatrics”, “Surveillance Protocols”, “TP53 mutation”, and “Toronto Protocol”, which were combined using Boolean operators (AND/OR) to optimize retrieval of relevant studies. Following article selection, sections of the review were distributed evenly among team members for independent analysis and drafting. Each section subsequently underwent peer review within the research team, and the final manuscript was approved after an editorial process involving two study members, with consensus achieved through discussion. 

Molecular pathogenesis of Li-Fraumeni syndrome

The molecular pathogenesis of LFS has become an important area of investigation because elucidating the mechanisms underlying malignant transformation may improve understanding of tumor development and identify potential targets for preventive and therapeutic interventions. LFS was first described in 1969 by Frederick P. Li and Joseph F. Fraumeni Jr. in the Annals of Internal Medicine. Their report described familial clustering of childhood soft-tissue sarcomas, early-onset breast cancer, and other malignancies among four affected families. Most of the sarcomas occurred before 15 years of age, and the pattern of malignancies was consistent with autosomal dominant inheritance [1].

The principal molecular driver of LFS is a germline pathogenic or likely pathogenic variant in TP53, located on chromosome 17p13.1. The gene encodes p53, a sequence-specific tumor-suppressor transcription factor that regulates cellular responses to genomic and oncogenic stress [2,10]. Under unstressed conditions, p53 is maintained at low intracellular concentrations, predominantly through MDM2-mediated ubiquitination and proteasomal degradation. Following cellular stress, including DNA damage, hypoxia, oxidative stress, and oncogene activation, regulatory modifications disrupt the interaction between p53 and MDM2, allowing p53 to accumulate and become transcriptionally active [11].

Activated p53 coordinates several protective responses that preserve genomic stability. It promotes cell-cycle arrest through transcriptional activation of CDKN1A, which encodes p21. The p21 protein inhibits cyclin-dependent kinase activity and restricts progression through the G1/S cell-cycle checkpoint [12]. P53 also participates in DNA-damage repair through transcriptional targets such as GADD45 and through regulatory effects on homologous recombination and non-homologous end joining [11,13]. When cellular damage is severe or cannot be repaired, p53 may promote apoptosis through transcriptional activation of pro-apoptotic genes, including BAX, PUMA, NOXA, and death-receptor pathway components such as FAS [14]. Through these responses, p53 limits the survival and propagation of cells containing potentially oncogenic genomic abnormalities. The roles of p53 in cell-cycle control, DNA repair, senescence, and apoptosis are well established.

In individuals with LFS, germline pathogenic TP53 variants impair one or more of these tumor-suppressive functions. Many pathogenic variants are missense substitutions involving the DNA-binding domain of p53, although pathogenic variants may also occur in other functional regions of the gene [2,4]. Mutant p53 proteins may contribute to tumorigenesis through loss of normal tumor-suppressor function, dominant-negative interference with residual wild-type p53, or acquisition of oncogenic gain-of-function properties [15]. In a dominant-negative mechanism, mutant and wild-type p53 proteins can assemble within the same tetramer, reducing the transcriptional activity of the remaining wild-type protein. Certain mutant p53 proteins may also acquire functions that promote cellular proliferation, invasion, genomic instability, or resistance to apoptosis [15].

Malignant transformation may be further facilitated by loss or functional inactivation of the remaining wild-type TP53 allele. Loss of heterozygosity has been observed in LFS-associated experimental and tumor models, although TP53-associated tumorigenesis should not be presented as requiring an identical classic two-hit mechanism in every tumor because dominant-negative and gain-of-function effects may contribute before or independently of complete loss of the wild-type allele [15,16]. The biological consequences of TP53 dysfunction vary according to the specific variant, the affected tissue, developmental context, and additional somatic alterations. These factors likely contribute to the heterogeneous tumor spectrum and variable penetrance observed among individuals with germline TP53 variants.

Cancer spectrum and natural history in the pediatric population

Li-Fraumeni syndrome is an important hereditary cause of malignancy in children and adolescents. Cancer risk begins in infancy and extends throughout life, although penetrance varies according to sex, age, TP53 variant type, and family ascertainment. The characteristic pediatric tumor spectrum includes adrenocortical carcinoma, soft-tissue and bone sarcomas, central nervous system tumors, and selected hematologic malignancies [4,17]. Population-based estimates should be interpreted cautiously because reported childhood cancer risks differ according to cohort composition and the pathogenicity and functional characteristics of the underlying TP53 variant.

Hematologic malignancies represent a less common but clinically important component of the LFS tumor spectrum. Germline TP53 variants are particularly associated with low-hypodiploid acute lymphoblastic leukemia, a biologically distinct ALL subtype characterized by marked aneuploidy and frequent disruption of TP53 signaling [18,19]. Genomic studies have demonstrated TP53 alterations in most childhood low-hypodiploid ALL cases, with germline variants accounting for a substantial proportion of pediatric cases [19]. Loss of heterozygosity involving the remaining wild-type TP53 allele may contribute to biallelic TP53 inactivation in leukemic cells [19]. Myeloid neoplasms have also been reported in individuals with LFS and may arise as primary malignancies or after exposure to chemotherapy or radiotherapy used to treat a previous cancer [18].

Rhabdomyosarcoma is the most common soft-tissue sarcoma of childhood and is among the sarcomas associated with germline TP53 variants [20,21]. The association is particularly notable in children with anaplastic rhabdomyosarcoma. In a retrospective series of children with rhabdomyosarcoma arising in the setting of germline TP53 mutations, anaplastic histology was identified in most evaluable tumors, supporting consideration of germline TP53 testing in children with this histologic pattern [22]. However, anaplastic morphology is not specific to LFS and should be interpreted together with age at diagnosis, personal cancer history, family history, and germline genetic testing.

At the molecular level, TP53 alterations occur more frequently in fusion-negative rhabdomyosarcoma than in fusion-positive disease and are generally associated with adverse biologic features [20,21]. Loss of normal p53 activity may impair cell-cycle control, apoptosis, and differentiation within mesenchymal precursor cells, thereby contributing to sarcoma development [23]. Experimental models have further demonstrated that mutant p53 and complete p53 loss can exert different effects on rhabdomyosarcoma initiation and progression. In a murine model of pleomorphic rhabdomyosarcoma, mutant p53 cooperated with oncogenic KRAS and produced biologic effects distinct from those observed with simple loss of p53 [24].

Adrenocortical carcinoma is rare in the general pediatric population but is a characteristic LFS-associated childhood malignancy. In a Children’s Oncology Group cohort, germline TP53 variants were identified in 44 of 88 tested children with adrenocortical carcinoma [6]. The prevalence of germline TP53 variants varies geographically and is particularly high in southern and southeastern Brazil because of the founder variant TP53 p.Arg337His [6,25]. This variant occurs within the p53 oligomerization domain and exhibits pH-dependent functional instability. Its strong association with childhood adrenocortical tumors may reflect both its biochemical characteristics and the developmental and metabolic environment of the adrenal cortex [25].

Central nervous system tumors are also well recognized within the LFS spectrum. Choroid plexus carcinoma is a rare, aggressive pediatric brain tumor with a particularly strong association with germline TP53 variants [26,27]. Published series suggest that approximately one-third to one-half of children with choroid plexus carcinoma may carry a germline TP53 variant, although estimates vary according to the cohort and geographic population [26-28]. Because of this association, germline TP53 testing should be considered in every child diagnosed with choroid plexus carcinoma, regardless of family history [2,28]. Diagnosis and classification rely on histopathologic and molecular evaluation; germline testing is performed to establish an underlying cancer-predisposition syndrome rather than to distinguish carcinoma from benign choroid plexus papilloma. The association between CPC and germline TP53 alterations is supported by both cohort data and subsequent reviews.

Other LFS-associated central nervous system malignancies include gliomas and medulloblastomas [4,17]. TP53-associated medulloblastomas most commonly occur within the sonic hedgehog molecular subgroup and may demonstrate large-cell or anaplastic histology. Germline TP53 alterations in sonic hedgehog medulloblastoma are associated with an increased risk of treatment failure and poor survival, making recognition of the underlying predisposition clinically important [29]. The original manuscript’s broader statement that all medulloblastomas with TP53 mutations have uniformly poor outcomes has therefore been narrowed to reflect the molecularly defined subgroup in which this association is best established.

Comparison of existing surveillance strategies

In 2000, Chompret and colleagues proposed clinical criteria intended to identify individuals and families in whom germline TP53 testing should be considered [30]. Unlike the original classic LFS criteria, which required a characteristic family history, the Chompret criteria allowed testing on the basis of an individual’s tumor type, age at diagnosis, and occurrence of multiple primary malignancies. The criteria have subsequently been modified as additional genotype-phenotype evidence has accumulated, progressively reducing reliance on a strongly positive family history [2,28].

The original Chompret criteria were based on childhood cancers and cancer patterns observed among TP53 mutation carriers [30]. Subsequent revisions broadened testing indications to include individuals with multiple primary tumors, characteristic rare tumors, and very early-onset breast cancer, even in the absence of a conventional LFS pedigree [2,28]. Children with adrenocortical carcinoma, choroid plexus carcinoma, and embryonal anaplastic-subtype rhabdomyosarcoma warrant particular consideration for germline TP53 evaluation because these tumors may be associated with pathogenic TP53 variants despite an unremarkable family history [2,22,28].

The association between anaplastic rhabdomyosarcoma and germline TP53 variants has influenced recommendations regarding genetic evaluation but should not be described as a formal universal addition to the Chompret criteria in 2015. Hettmer et al. recommended consideration of germline TP53 testing in children with anaplastic rhabdomyosarcoma, while subsequent COG-focused reviews have emphasized that TP53 alteration status may provide important prognostic and risk-stratification information in rhabdomyosarcoma [20,22]. The manuscript should therefore distinguish between formal Chompret criteria and tumor-specific proposals arising from rhabdomyosarcoma studies.

The multimodal surveillance approach commonly referred to as the Toronto Protocol was evaluated prospectively by Villani and colleagues beginning with their 2011 study and subsequently through extended follow-up published in 2016 [8,9]. The protocol combined regular physical examination, biochemical testing, abdominal and pelvic ultrasonography, brain MRI, whole-body MRI, and age- and sex-specific surveillance. The precise tests and intervals varied according to age and target malignancy [8,9]. The 2016 follow-up demonstrated that surveillance was associated with earlier cancer detection and improved survival, providing the principal prospective evidence supporting intensive surveillance in germline TP53 variant carriers [9].

Contemporary recommendations have refined the Toronto approach rather than reproducing it unchanged. The 2020 ERN GENTURIS guideline recommends germline TP53 testing for individuals who meet the modified Chompret criteria and for selected patients with tumors strongly associated with heritable TP53-related cancer syndromes [2]. In children, the guideline recommends clinical examination every six months, abdominal ultrasonography every six months, annual whole-body MRI, and annual brain MRI, with additional testing guided by clinical findings and previous treatment exposure [2]. Germline testing is also recommended or considered for selected children with low-hypodiploid ALL, choroid plexus carcinoma, adrenocortical carcinoma, and sonic hedgehog medulloblastoma, depending on the clinical and molecular context [2].

The original manuscript stated that children with “jaw osteosarcoma” are specifically recommended for testing under the ERN guideline. That statement is not clearly supported by the guideline and has been removed. Similarly, the recommendation for testing children with malignancy from southern or southeastern Brazil is more accurately linked to the high prevalence of the TP53 p.Arg337His founder variant and should not be interpreted as meaning that every child from the region with any cancer automatically fulfills the same testing criterion [2,25].

Compared with ERN GENTURIS, Toronto-derived and AACR recommendations generally use shorter pediatric intervals for some clinical assessments and abdominal imaging. The 2025 AACR consensus recommends comprehensive physical examination and abdominal and pelvic ultrasonography every three to four months during childhood, annual whole-body MRI, and annual brain MRI, with surveillance adapted according to age, sex, previous cancer history, and organ-specific risk [7]. In contrast, the ERN GENTURIS guideline generally recommends six-month intervals for pediatric physical examination and abdominal ultrasonography [2]. These differences should be presented as variations between guidelines rather than attributing every recommendation to a single uniform “Toronto Protocol.”

Breast surveillance recommendations should likewise be attributed to contemporary consensus guidelines rather than solely to the original Toronto prospective study. Current recommendations emphasize breast awareness beginning in adolescence and annual contrast-enhanced breast MRI beginning in early adulthood, while mammography is generally minimized or carefully considered because it involves ionizing radiation [2,7]. The original sentence stating that the Toronto Protocol uniformly recommends both annual breast MRI and mammography, with ultrasound used only for additional information, was therefore too broad and has been replaced.

Emerging therapeutic strategies in Li-Fraumeni syndrome

As understanding of Li-Fraumeni syndrome and germline TP53-associated cancer risk continues to evolve, contemporary surveillance recommendations have become increasingly age- and organ-specific. In 2025, the American Association for Cancer Research updated its surveillance recommendations for individuals with LFS, emphasizing that screening intensity and modality should reflect changes in tumor risk across the lifespan [7]. In children, these recommendations include comprehensive physical examinations and abdominal and pelvic ultrasonography every three to four months, together with annual whole-body MRI and brain MRI. Surveillance intervals become less frequent for selected assessments in adulthood and are supplemented by sex- and organ-specific screening [7].

The updated recommendations also address the increasing identification of asymptomatic germline TP53 variant carriers through multigene panel testing. These individuals, sometimes described as “previvors,” have not developed cancer but remain at substantial lifelong risk, creating a need for prospective evidence regarding the benefits, burdens, and optimal intensity of surveillance in this population [31]. Lifelong screening may also create considerable psychological stress, particularly during transition from pediatric to adult care. Consequently, surveillance programs should incorporate genetic counseling, psychosocial support, and shared decision-making in addition to imaging and laboratory evaluation [7,31].

Minimizing exposure to ionizing radiation remains an important principle in the management of individuals with germline TP53 variants. Impaired p53-mediated responses to DNA damage may increase susceptibility to radiation-associated malignancies, although radiotherapy should not be regarded as absolutely contraindicated when it is necessary for curative treatment. Treatment decisions should instead balance oncologic benefit against the potential risk of subsequent malignancy, and non-ionizing imaging modalities should be favored for surveillance whenever clinically appropriate [32]. Accordingly, magnetic resonance imaging and ultrasonography are generally preferred over repeated computed tomography or other ionizing-radiation-based surveillance studies [2,7,32].

Emerging molecular technologies may eventually complement conventional imaging. Cell-free DNA-based liquid biopsy has demonstrated the ability to identify cancer-associated signals in individuals with LFS, including signals detected before or in conjunction with conventional clinical evaluation [33]. In the reported study, multimodal cell-free DNA analysis demonstrated promising diagnostic performance, supporting its potential role as an adjunct to surveillance [33]. However, these findings require validation in larger prospective cohorts before liquid biopsy can replace or substantially reduce established imaging-based surveillance.

Therapeutic development in LFS is especially challenging because TP53 is a tumor-suppressor gene rather than a conventional activating oncogene. Loss or alteration of p53 affects multiple interconnected cellular processes, including cell-cycle regulation, apoptosis, DNA repair, metabolism, senescence, and genomic stability. Consequently, TP53 dysfunction does not create one universal therapeutic vulnerability that can be targeted across all tumors and variants [34,35].

Current investigational strategies can be broadly divided into three categories: restoration of mutant p53 function, exploitation of vulnerabilities created by TP53 dysfunction, and pharmacologic prevention of malignant transformation.

One approach involves restoring wild-type-like activity to mutant p53 proteins. Eprenetapopt, also known as APR-246, is a mutant p53-reactivating compound that is converted intracellularly to methylene quinuclidinone, a reactive metabolite capable of binding cysteine residues within mutant p53 and promoting conformational stabilization [35]. Although eprenetapopt has undergone clinical investigation in several TP53-mutated malignancies, it has not been approved specifically for cancer prevention or treatment in individuals with germline TP53 variants.

Rezatapopt, formerly designated PC14586, represents a more mutation-specific approach. It is an orally bioavailable small molecule designed to bind the structural pocket created by the TP53 Y220C substitution, stabilize the mutant protein, and restore wild-type-like transcriptional activity [36]. This strategy illustrates the potential for variant-specific treatment but is applicable only to tumors harboring the Y220C alteration.

Arsenic trioxide has also demonstrated mutant p53-reactivating activity. Structural studies identified a cryptic allosteric binding site within the p53 DNA-binding domain through which arsenic can stabilize selected structural mutants and restore transcriptional activity [37]. Subsequent work demonstrated that responsiveness varies among TP53 variants according to their intrinsic structural and biochemical properties [38]. These findings suggest that arsenic-based rescue may be feasible for selected mutations but should not be generalized to all germline or somatic TP53 variants.

Zinc metallochaperones represent another strategy for restoring mutant p53 function. Some structural TP53 variants impair zinc binding, destabilizing the DNA-binding domain and preventing normal transcriptional activity. Thiosemicarbazone-based compounds can function as zinc metallochaperones by increasing intracellular zinc availability and promoting the refolding of selected zinc-deficient p53 mutants [39]. ZMC1 has similarly been shown to transport zinc into cells and restore wild-type-like conformation and activity in susceptible mutant p53 proteins [40]. Additional biochemical work has demonstrated that zinc availability influences the folding landscape of p53 and may permit reactivation of structurally diverse mutants under appropriate conditions [41]. These compounds remain investigational and are unlikely to be uniformly effective across all TP53 variants.

Parallel approaches seek to exploit cellular vulnerabilities created by p53 deficiency. TP53-mutant tumors may become more dependent on alternative cell-cycle checkpoint and DNA-damage-response pathways, including WEE1, ATR, and CHK1 signaling. Inhibition of these pathways has demonstrated antitumor activity in preclinical models and early clinical studies of TP53-altered cancers [34,35]. However, these approaches have not yet been established as LFS-specific therapies, and their efficacy may depend on tumor type, coexisting molecular alterations, and the specific functional consequences of the TP53 variant.

Gene-dosage modification represents a more preventive experimental approach. Preclinical studies in LFS models have suggested that the introduction or preservation of additional functional p53 activity may delay tumor development and prolong survival [42]. Although this concept supports the biological rationale for p53-restorative prevention, gene-based prophylactic therapy remains far from routine clinical application.

Given the difficulty of directly targeting every pathogenic TP53 variant, increasing attention has shifted toward modifying the biological environment that precedes malignant transformation. The “pre-cancer niche” model proposes that germline TP53 dysfunction promotes chronic oxidative stress, inflammation, metabolic abnormalities, altered angiogenesis, and immune dysregulation, thereby creating conditions that facilitate tumor initiation [43]. This framework has encouraged investigation of repurposed agents such as metformin, aspirin, statins, propranolol, sirolimus, and bisphosphonates as potential cancer-interception strategies, although most currently lack disease-specific clinical evidence [43].

Among these agents, metformin has the strongest LFS-specific preclinical and early clinical support. In an LFS mouse model, inhibition of mitochondrial respiration reduced oxidative metabolism, delayed tumor development, and prolonged cancer-free survival [44]. A subsequent pilot study in individuals with LFS demonstrated that metformin was generally tolerable and produced measurable metabolic effects, although the study was not designed to establish cancer-preventive efficacy [45]. These findings led to the Metformin in Li-Fraumeni Syndrome trial, a randomized cancer-prevention study evaluating metformin in adults with LFS undergoing annual MRI surveillance [46].

Although mutant p53 reactivation, synthetic-lethal targeting, gene-dosage modification, and metabolic intervention are scientifically promising, none has yet demonstrated sufficient efficacy and safety to become standard preventive therapy for germline TP53 variant carriers. At present, structured surveillance remains the principal evidence-based strategy for reducing LFS-associated mortality, while targeted and preventive treatments remain investigational.

Discussion

The introduction of structured surveillance has substantially changed the clinical management of Li-Fraumeni syndrome by shifting the emphasis from treatment of clinically advanced malignancy toward early detection. The original Toronto surveillance studies and subsequent recommendations from ERN GENTURIS and the AACR support a multimodal approach incorporating regular clinical assessment, whole-body MRI, dedicated brain MRI, abdominal and pelvic ultrasonography, and age- and organ-specific investigations [2,7-9]. The extended prospective Toronto study demonstrated that individuals undergoing surveillance had improved overall survival compared with those who did not participate in structured screening, providing the strongest available evidence in support of intensive LFS surveillance [9].

Despite this benefit, implementation of lifelong surveillance presents significant practical and psychosocial challenges. Children and families may require frequent imaging, laboratory testing, multidisciplinary appointments, genetic counseling, and evaluation of incidental abnormalities. Whole-body MRI may identify findings that ultimately prove benign or clinically insignificant, leading to additional imaging, invasive procedures, expense, and anxiety [7,9]. These limitations are particularly relevant in children, who may require sedation for MRI and must continue surveillance across multiple developmental stages.

The psychological effects of surveillance are not uniformly negative. Patient-reported outcomes from the Li-Fraumeni Education and Early Detection program indicate that surveillance may provide reassurance, improve perceived control, and reduce some uncertainty associated with cancer risk, although anxiety and distress remain important concerns for a subset of participants [47]. These findings support the integration of psychosocial assessment, genetic counseling, and shared decision-making into routine surveillance rather than treating screening as a purely radiologic or laboratory-based intervention.

Important evidentiary limitations remain. Most LFS surveillance recommendations are derived from prospective observational studies, retrospective cohorts, and expert consensus rather than randomized controlled trials [2,7-9]. Randomization to surveillance versus no surveillance would also be ethically and practically difficult given the survival benefit already observed in prospective cohorts. Nevertheless, uncertainty persists regarding the optimal frequency of individual studies, the value of some biochemical tests, the long-term effects of repeated imaging, and the extent to which surveillance should be modified according to genotype, previous malignancy, treatment exposure, sex, or family history.

These uncertainties are especially important in pediatric patients because the childhood tumor spectrum differs substantially from that of adults. Children with LFS are particularly susceptible to adrenocortical carcinoma, sarcomas, brain tumors, and selected hematologic malignancies, whereas breast cancer and several other solid tumors become increasingly relevant later in life [4,6,17-29]. Pediatric surveillance must therefore balance early tumor detection against the burdens of repeated imaging, sedation, false-positive findings, and disruption of school and family life.

Further research should focus on prospective multicenter surveillance cohorts with standardized reporting of cancer-detection rates, stage at diagnosis, false-positive findings, invasive follow-up procedures, psychosocial outcomes, survival, and cost-effectiveness. Greater attention should also be directed toward genotype-phenotype associations. Different TP53 variants may produce variable penetrance, age at onset, dominant-negative activity, and tumor spectrum, suggesting that future surveillance may be increasingly individualized rather than uniformly applied to all germline variant carriers [4,15,16].

Molecular biomarkers may eventually improve the sensitivity and specificity of early cancer detection. Cell-free DNA analysis has shown promising ability to detect cancer-associated signals in individuals with LFS [33]. However, liquid biopsy remains investigational and should currently be viewed as complementary to, rather than a replacement for, established imaging protocols. Prospective studies are required to determine whether it can reliably detect the diverse malignancies associated with LFS, reduce false-positive imaging, identify cancers at a clinically meaningful stage, and improve survival.

Therapeutic development remains another major unmet need. Investigational approaches include mutant p53 reactivation, variant-specific compounds such as rezatapopt, arsenic-mediated structural rescue, zinc metallochaperones, and inhibition of WEE1, ATR, or CHK1-dependent pathways [34-41]. These strategies are biologically compelling but have largely been evaluated in preclinical models, molecularly selected solid tumors, or non-LFS clinical populations. Their relevance to germline TP53 carriers must therefore be established separately, particularly because therapeutic response may vary according to the individual TP53 variant and tumor context.

Cancer-prevention strategies may ultimately be especially important in LFS because affected individuals face repeated and lifelong risks of primary malignancies. The pre-cancer niche model provides a conceptual framework for targeting inflammation, oxidative stress, mitochondrial metabolism, and other early carcinogenic processes before a tumor becomes clinically detectable [43]. Metformin is currently the most advanced disease-specific example: it delayed tumor formation in an LFS mouse model, demonstrated acceptable tolerability and metabolic activity in a human pilot study, and is now being evaluated prospectively in the MILI randomized trial [44-46]. However, metformin should remain investigational until the trial demonstrates whether metabolic changes translate into a clinically meaningful reduction in cancer incidence.

Overall, surveillance remains the foundation of clinical management for children and adults with LFS. The available evidence supports early initiation of age-specific screening, preferential use of non-ionizing imaging, and coordinated multidisciplinary follow-up. Future progress will depend on improved risk stratification, prospective validation of molecular detection technologies, development of variant-informed therapies, and adequately designed cancer-prevention studies. Integrating these advances with psychosocial and genetic support will be essential to improve outcomes without unnecessarily increasing the burden of lifelong surveillance.

## Conclusions

Li-Fraumeni Syndrome represents one of the most clinically significant hereditary cancer predisposition syndromes encountered in pediatric oncology, with germline TP53 mutations conferring a markedly increased lifetime risk of multiple early-onset malignancies. Over the past decade, substantial advances have been made in understanding the molecular biology underlying LFS, enabling improved characterization of tumor development and progression and informing more comprehensive surveillance strategies. The implementation of structured surveillance protocols, particularly the Toronto Protocol and subsequent international guideline refinements, has transformed clinical management by facilitating earlier cancer detection and improving survival outcomes in affected individuals. Nevertheless, considerable challenges remain, including the psychosocial burden of lifelong surveillance, the risk of false-positive findings, disparities in access to advanced imaging, and the absence of robust prospective pediatric studies to further optimize surveillance recommendations.

Although no targeted therapy has yet been approved specifically for individuals with LFS, significant progress has been made in the development of novel therapeutic approaches targeting mutant p53 reactivation, synthetic lethality, and pharmacologic cancer prevention. Emerging precision medicine strategies, together with advances in molecular diagnostics and genotype-directed risk stratification, offer considerable promise for the future management of this syndrome. Continuing international collaboration, conducting prospective multicenter research, and integrating translational discoveries into clinical practice will be essential to refine surveillance protocols, validate emerging therapeutic interventions, and ultimately improve long-term outcomes and quality of life for children and families living with Li-Fraumeni Syndrome.
